# Adipose depot gene expression and intelectin‐1 in the metabolic response to cancer and cachexia

**DOI:** 10.1002/jcsm.12568

**Published:** 2020-03-31

**Authors:** Janice Miller, Gillian Dreczkowski, Michael I. Ramage, Stephen J. Wigmore, Iain J. Gallagher, Richard J.E. Skipworth

**Affiliations:** ^1^ Clinical Surgery, Royal Infirmary of Edinburgh University of Edinburgh Edinburgh UK; ^2^ Faculty of Health Science and Sport University of Stirling Stirling UK

**Keywords:** Cancer cachexia, Adipose, Intelectin, Microarray, Genes

## Abstract

**Background:**

Cancer cachexia is a poorly understood metabolic consequence of cancer. During cachexia, different adipose depots demonstrate differential wasting rates. Animal models suggest adipose tissue may be a key driver of muscle wasting through fat–muscle crosstalk, but human studies in this area are lacking. We performed global gene expression profiling of visceral (VAT) and subcutaneous (SAT) adipose from weight stable and cachectic cancer patients and healthy controls.

**Methods:**

Cachexia was defined as >2% weight loss plus low computed tomography‐muscularity. Biopsies of SAT and VAT were taken from patients undergoing resection for oesophago‐gastric cancer, and healthy controls (*n* = 16 and 8 respectively). RNA was isolated and reverse transcribed. cDNA was hybridised to the Affymetrix Clariom S microarray and data analysed using R/Bioconductor. Differential expression of genes was assessed using empirical Bayes and moderated‐*t*‐statistic approaches. Category enrichment analysis was used with a tissue‐specific background to examine the biological context of differentially expressed genes. Selected differentially regulated genes were validated by qPCR. Enzyme‐linked immunosorbent assay (ELISA) for intelectin‐1 was performed on all VAT samples. The previously‐described cohort plus 12 additional patients from each group also had plasma I = intelectin‐1 ELISA carried out.

**Results:**

In VAT vs. SAT comparisons, there were 2101, 1722, and 1659 significantly regulated genes in the cachectic, weight stable, and control groups, respectively. There were 2200 significantly regulated genes from VAT in cachectic patients compared with controls. Genes involving inflammation were enriched in cancer and control VAT vs. SAT, although different genes contributed to enrichment in each group. Energy metabolism, fat browning (e.g. uncoupling protein 1), and adipogenesis genes were down‐regulated in cancer VAT *(P* = 0.043, *P* = 5.4 × 10^−6^ and *P* = 1 × 10^−6^ respectively). The gene showing the largest difference in expression was ITLN1, the gene that encodes for intelectin‐1 (false discovery rate‐corrected *P* = 0.0001), a novel adipocytokine associated with weight loss in other contexts.

**Conclusions:**

SAT and VAT have unique gene expression signatures in cancer and cachexia. VAT is metabolically active in cancer, and intelectin‐1 may be a target for therapeutic manipulation. VAT may play a fundamental role in cachexia, but the down‐regulation of energy metabolism genes implies a limited role for fat browning in cachectic patients, in contrast to pre‐clinical models.

## 1 Introduction

Cachexia is a complex metabolic syndrome associated with several chronic diseases, including cancer, that is characterised by loss of lean and adipose tissue^1^. Cachexia is a major burden for cancer patients, and it impacts negatively on their response to treatment, their quality of life, and survival. Although the consensus definition of cancer cachexia^2^ emphasises loss of lean tissue (particularly skeletal muscle) as a key factor in the diagnosis of the condition, the loss of adipose tissue in cancer cachexia is increasingly thought to play an important role.^3^, ^4^

In cancer, the loss of adipose tissue is driven by lipolysis^5^ rather than adipocyte apoptosis or necrosis. Mediators of lipolysis in cancer cachexia (CC) are largely unknown. In the past, members of our group have identified increased mRNA expression of zinc‐α2‐glycoprotein (ZAG), a proposed lipid mobilizing factor, in fat samples from CC patients; however, serum ZAG levels were unchanged from controls.^6^, ^7^ Microarray analysis has revealed that changes in the transcriptome of subcutaneous fat in CC are opposite to those seen in obesity, underlining the importance of lipolysis.^8^ Visceral adipose tissue is thought to be lost more rapidly than subcutaneous adipose tissue during cachexia, suggesting differential adipose depot‐dependent responses to the wasting process though this has not been consistently demonstrated in humans.^9^ Bioinformatic analysis of mRNA expression in visceral (omental) and subcutaneous adipose depots in noncachectic, obese endometrial cancer patients demonstrated 19 shared biological pathways, 18 of which were regulated in opposite directions between the fat depots.^10^ Recently, focus has concentrated on the concept of ‘fat–muscle crosstalk’ in CC.^1^ Notably, genetic ablation of lipolytic pathways in adipocytes protects against muscle mass loss in animal models of CC.^11^ Inhibition of lipolysis through genetic ablation of adipose triglyceride lipase or hormone sensitive lipase was shown to reduce muscle wasting.^11^ These data support the loss of visceral fat driven by lipolytic mechanisms as an early event in CC with a potential effect on skeletal muscle.

Global gene expression profiling is an effective method to examine regulatory pathways in patho‐physiological contexts.^12^ In our previous studies, microarray analysis of skeletal muscle biopsies taken at the time of resectional surgery for upper gastrointestinal (GI) cancer revealed an 83‐gene signature that was able to identify patients with >5% weight loss.^13^ In further studies, a transcriptomic comparison of sequential muscle biopsies (at surgery and 8 months post‐surgery) from upper GI cancer patients revealed 1868 regulated genes associated with cancer and weight loss.^14^ Contrary to expectation, the vast majority (94%) of genes were down‐regulated. Category analysis of the differentially expressed genes showed that both anabolic and catabolic process gene expression was suppressed in cancer. Furthermore, there was a lack of substantive overlap with transcriptomic signatures from endurance training, strength training, ageing, or simple dieting. Highly enriched categories included lipid oxidation, fatty acid metabolism, and peroxisome pathways, implying a role for fat–muscle crosstalk in CC.^14^ A better understanding of the physiological effects of cancer on adipose tissue might open new therapeutic avenues for the amelioration of both fat and skeletal muscle wasting resulting in improved outcomes.

In the current study, we examined the global transcriptome of VAT and SAT depots in patients with confirmed CC, according to the diagnostic consensus definition [>2% weight loss and low muscularity on a computed tomography (CT) scan; *n* = 8] compared with weight stable cancer (CWS) patients (*n* = 8), as well as healthy controls undergoing donor nephrectomy (*n* = 8). These data provide the first examination of global gene expression in these two fat depots in CC and provide a basis for hypothesis generation and exploring treatments for CC.

## 2 Methods

### 2.1 Study participants

Patients undergoing surgical resection with curative intent for upper GI cancer (oesophageal, gastric, or pancreatic) were recruited via the regional multidisciplinary team meeting. Healthy controls undergoing surgery for renal transplant donation were approached at their pre‐assessment appointment. Participants had to be over the age of 18 and able to give written, informed consent. The study was approved by the Lothian regional ethics committee (IRAS ID: 190214) and conformed to the declaration of Helsinki. Control and CWS patients were excluded if they were found to be sarcopenic on CT scan. Patient demographics are described in terms of median (interquartile range). A Kruskal Wallis test was used to compare groups.

### 2.2 Adipose tissue biopsies

Following surgical skin incision under general anaesthesia, biopsies of subcutaneous fat were taken from all patients at the start of the operation. Visceral fat was sampled from the greater omentum in cancer patients and perinephric fat from the transplant donors after the kidney had been excised. All samples were snap frozen in liquid nitrogen and stored at −80°C until analysis.

### 2.3 Plasma samples

Venous blood samples were taken at induction of anaesthesia from all patients. Samples were centrifuged at 4°C at 15 000 *g* for 15 min. Plasma was then withdrawn and divided into 1 mL aliquots and frozen at −80°C until use.

### 2.4 Muscle cross‐sectional area

Skeletal muscle, visceral, and subcutaneous adipose cross‐sectional areas were calculated from routine staging CT scans performed prior to any surgical intervention or neoadjuvant chemotherapy. Digitally stored CT images completed with a spiral CT were analysed using slice‐O‐matic and ABACS software (Voronoi Health Analytics, Canada). The cross‐sectional area for muscle, visceral, and subcutaneous adipose tissue was normalized for stature (cm^2^/m^2^) at the level of the third lumbar vertebrae [skeletal muscle index (SMI), visceral adipose index, and subcutaneous adipose index, respectively]. SMI cut‐offs for low muscularity were taken from previous reference studies of cancer patients by Martin *et al*.^15^ Cachexia was classified according to the consensus definition by Fearon *et al*. (specifically >2% weight loss with low muscularity).^2^ This specific definition of CC was used as it was shown to be associated with confirmed histological muscle fibre wasting in our previous studies.^16^

### 2.5 Transcriptomic analysis

Aliquots of adipose tissue were homogenised in a Qiazol reagent (Qiagen, UK) using the BeadBeater instrument with 1.5 mm ceramic beads (Qiagen, UK). Tissue aliquots were shaken at 5000 Hz for 30 s with 30 s cooling on ice. This process was repeated three times or until no gross debris was visible. Samples were then spun at 2800 g for 15 mins at 4°C and the supernatant collected into fresh Eppendorf tubes. RNA was isolated from adipose tissue samples using the Qiagen RNeasy lipid tissue kit (Qiagen, UK) following the manufacturer's directions. RNA was quantified using the Denovix DS11 FX+ spectrophotometer (Denovix, UK). RNA quality was examined using the RNA iQ assay and the Qubit 4 instrument (Thermo Fisher, UK). All samples returned quality scores ≥7.7. Complementary DNA was generated using the GeneChip WT Plus Reagent kit (Thermo Fisher, UK) as per directions. Samples were hybridised to the Clariom S GeneChip microarray (Thermo Fisher, UK) as per manufacturers directions. After washing and staining, images were captured, and CEL files generated using the GeneChip Scanner 3000 (Thermofisher UK). The raw CEL files can be accessed at the Gene Expression Omnibus (GSE131835).

### 2.6 Quantitative polymerase chain reaction

Total RNA was extracted as above, and cDNA was prepared using the High Capacity cDNA Reverse Transcription kit (Applied Biosystems, cat no. 4368814) with 500 ng total RNA input for each reaction. Quantitative PCR (qPCR) was carried out using Luminaris Hi Green Low ROX qPCR Master Mix (Thermo Fisher UK, Cat no. K0974) 5 μL, 0.3 μL forward and reverse primer at 10 μm each and 3.4 μL ddH_2_O. Primers were selected from the Primer Bank resource^17^ except for actin beta and GAPDH. These latter primers were designed to span exons using the Primer‐BLAST resource.^18^ We selected primers to cover genes involved in inflammation (ACVR2A1, IL6, and IL18), adipogenesis (LEPR, STAT5A, and PDCD4), adipose browning (UCP1, PPARGC1A, and PRDM16) as well as genes that demonstrated a substantial statistically significant fold change by microarray data (ITLN1, ALOX15, and PPARA). We also profiled five genes to use as reference values in PCR data normalization as recommended by the MIQE guidelines.^19^ PCR reactions were carried out on the LightCycler 480 Instrument with the following protocol; 50°C 2mins (uracil–DNA–glycosylase treatment), 95°C 10 min (DNA denaturation) and 40 cycles of 95°C 15 s and 60°C 60s before a final melting curve step. Samples were run in triplicate on 384‐well plates. Analysis of qPCR data was carried out using the NormqPCR package in R/Bioconductor.^20^ The geNorm method^21^ was used to identify the four most stable control genes from actin beta, GAPDH, YWHAZ, POLR2A, and PSMB2, and the geometric mean of these was used to normalise the cycle threshold values for the remaining genes. Relative gene expression was calculated using the delta–delta Cq method (14). Primers used are shown in the Supporting Information.

### 2.7 Enzyme‐linked immunosorbent assay

Intelectin‐1 (Itln1) concentration was assessed in the VAT and plasma using an enzyme‐linked immunosorbent assay [(ELISA) (Amsbio EH0564); sensitivity <46.875 pg/mL; reference range: 78.125–5000 pg/mL]. A further 36 patients were recruited to increase the total of plasma samples to 20 for each group. Plasma was diluted to a 1 to 10 concentration and assayed in duplicates according to the manufacturer's instructions. Intra‐assay and interassay coefficients of variation were <8 and <10%, respectively.

Tissue ELISA was carried out on homogenised VAT tissue. Adipose tissue was homogenised in RIPA buffer with added protease inhibitors (Thermo Fisher, UK). Tissue aliquots were added to 1.5 mL PowerBead tubes with 1.4 mm ceramic beads (Qiagen cat no 13113) and shaken at 5000 r.p.m. for 20 s with 10 s rest on ice three times using the MagNALyser instrument (Roche, UK). The homogenate was centrifuged at 5000 r.p.m. for 15 min at 4°C, and the aqueous phase beneath the lipid cake and above the cell pellet was removed to a separate tube. Aliquots were diluted 1:20 in PBS and protein content quantified using the Qubit 4 Protein Assay (Thermo Fisher). The same ELISA kit was used for VAT and plasma (Amsbio EH0564).

### 2.8 Statistical analysis

**Figure 1:**
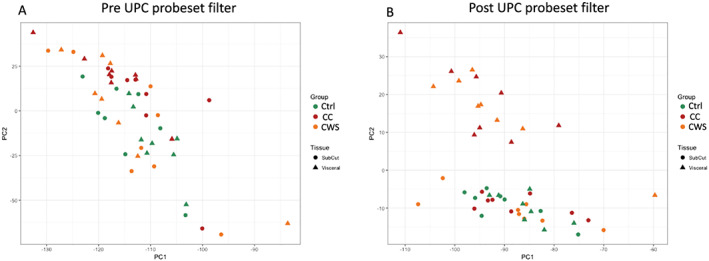
Scatterplots showing separation of adipose depot and group samples by principle components analysis. Before (A) and after (B) removal of non/low expressed genes using the Universal exPression Code (UPC) (Piccolo, 2013) generated filter. CC, cancer cachexia; Ctrl, healthy control; CWS, cancer weight stable.

The microarray data were analysed using R/Bioconductor using an updated chip definition file from the Brainarray resource^22^ based on Ensembl gene definitions (ENSG version 22). Raw microarray data were normalized and filtered using the SCAN‐UPC method.^23^ Expression intensity and UPC filters were set at 0 and 0.5 respectively. Use of these filters suggested 49% of the genes with probes in the microarray chip definition file were not expressed in adipose tissue. This is in line with our previous experience using Affymterix GeneChip microarrays. Filtering also improved the clustering of samples by principle components analysis (*Figure* 1). Changes in differential expression were examined using empirical Bayes and a moderated *t* statistic implemented in the limma package^24^ with subject identifier as a blocking factor in linear modelling to account for the repeated measures structure (see section Additive Models and Blocking in the limma manual). Statistical significance was taken as a false discovery rate <5%. Category enrichment analysis was carried out using the camera algorithm^25^ and the Broad Institute curated Hallmark Genesets collection.^26^ A false discovery rate <5% was taken to indicate significant enrichment of a category.

The qPCR data were analysed using the R/Bioconductor NormqPCR package.^20^ The expression data was modelled with a mixed effects linear model using the lme4 R package^27^ with group, fat depot, and the interaction between these as fixed factors and subject identifier as a random factor to account for pairing. Post hoc testing of interaction or main effects was carried out using the emmeans^28^ package.

Itln1 concentrations were calculated from raw ELISA data in R using the nCal package.^29^ After subtraction of blank values as recommended by the kit manufacturer a 5‐PL model was fit to the data and concentrations back calculated. Statistical analysis was carried out on log transformed data. Tissue ELISA data were analysed using a linear model with log Itln1 concentration modelled by group. For the plasma ELISA data, the log Itln1 concentrations from Assays 1 and 2 were combined and modelled with a mixed effects model using the lme4 package^27^ with assay as a random factor. Post hoc testing was carried out using the emmeans package.^28^ Statistical significance was set at *P* ≤ 0.05.

## 3 Results

### 3.1 Patient characteristics

Twenty‐four patients were included in the study with eight subjects in each of the control, CWS, and CC groups. Patient demographics and anthropometric details are shown in *Table* 1. Median weight loss was significantly higher (12.5%, interquartile ratio 6.87–15.5, *P* < 0.001), and SMI was significantly lower in the CC group (*P* = 0.002). Control patients were younger (median 51 years compared with 60 in the CWS group and 70 in the CC group, *P* = 0.002). Final pathology results for all patients can be seen in the Supporting Information.

**Table 1:** Demographic and selected clinical data for study subjects

Group (*n* = 8)	Control	Cachexia	Weight stable	P value
Male:Female	4:4	5:3	6:2	/
Age (years)	51 (48–55)	70 (64–77)	60 (57–64)	0.002
BMI	25.5 (23.75–28.25)	24 (20.25–29.25)	31.5 (23–40)	0.227
%weight loss	0	12.5 (6.87–15.5)	0	<0.001
SMI	48.30 (44.32–53.88)	39.99 (38.24–41.13)	55.52 (48.40–64.22)	0.002
VATI	20.97 (11.05–43.06)	23.3 (5.82–38.76)	55.52 (48.40–64.22)	0.196
SATI	50.32 (29.46–63.43)	50.75 (20.15–98.77)	70.35 (34.04–120.67)	0.605
OACC:OSCC:GACC	N/A	6:0:2	6:1:1	

Median (Interquartile range) values are presented.

BMI, body mass index; SMI, skeletal muscle index; VATI, Visceral adipose index; SATI, Subcutaneous adipose index

### 3.2 Microarray analysis

**Figure 2:**
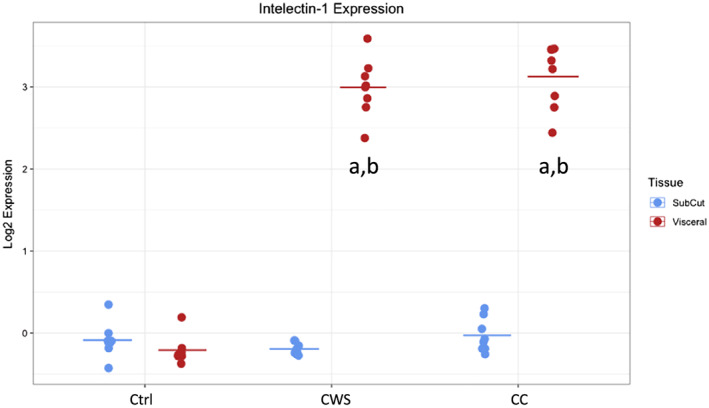
Intelectin‐1 mRNA expression values (log2 transformed) from the microarray data. CC, cancer cachexia; Ctrl, healthy controls; CWS, cancer weight stable. a = significantly different from paired subcutaneous values; b = significantly different from control visceral values.

Filtering the microarray data as recommended by the SCAN‐UPC authors^23^ led to the identification of 8679 probesets (approximately 49%) as unlikely to be expressed in adipose tissue. These were removed from the data set prior to further analysis to improve statistical power. Principle components analysis indicated a substantive difference in VAT vs. SAT transcriptome profiles in the presence of cancer (*Figure* 1). We first examined mRNA expression differences between the SAT and VAT depots within each group. In the CC group, we found 2101 probesets significantly regulated between the fat depots with fold changes ranging from eight‐fold up‐regulated to two‐fold down‐regulated. In the CWS group, we found 1722 probesets significantly regulated with fold changes ranging from nine‐fold up‐regulated to 2.5‐fold down‐regulated. In the control group, we found 1659 probesets differentially regulated with fold changes from 4.6‐fold up‐regulated to 2.3‐fold down‐regulated. These data indicate that SAT and VAT depots have substantial mRNA expression differences. In addition, the number of differentially expressed probesets is higher in the cancer groups and highest in the CC group (table 2). Comparing VAT between the groups, we found 2200 probesets regulated (10‐fold up to 2.3‐fold down) between CC and CWS, and 1253 probesets regulated (nine‐fold up to 2.3‐fold down) between CC and control. No probesets met the statistical cut‐off for significance in VAT between the CC and CWS groups. ITLN1, the gene which encodes for the novel adipocytokine Itln1, was the most up‐regulated gene with a 10‐fold increase in CC vs. control and a nine‐fold increase in CWS vs. control (*Figure* 2). Intelectin‐2 was also significantly increased in these comparisons. SAT demonstrated no significant differences in any comparison between CC, CWS, and control. Full details of regulated mRNA are available in the Supporting Information. These data demonstrate that whilst the transcriptional profile of SAT is not sensitive to the presence of cancer or CC in our cohort, there are substantial differences in VAT mRNA expression in the presence of cancer.

**Table 2:** Number of genes regulated in the microarray data with FDR < 5% in each within and between group comparison

	No. regulated genes (FDR < 5%)
VAT vs. SAT in group comparison	
CC VAT vs. SAT	2101
CWS VAT vs. SAT	1722
Control VAT vs. SAT	1659
VAT between group comparisons	
CC vs. control	2200
CWS vs. control	1253
CC vs. cWS	0^a^
SAT between group comparisons	
CC vs. Control	0^b^
CWS vs. Control	0^c^
CC vs. CWS	0^d^

CC, cancer cachexia; CWS, cancer weight stable; FDR, false discovery rate; SAT, subcutaneous adipose tissue; VAT, visceral adipose tissue.

a lowest adjusted *P* value = 0.7

b lowest adjusted *P* value = 0.1

c lowest adjusted *P* value = 1

d lowest adjusted *P* value = 0.5.

### 3.3 Geneset enrichment analysis

**Figure 3:**
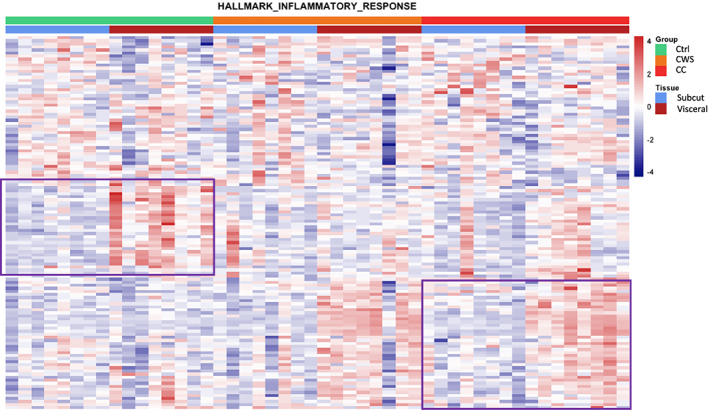
Heatmap of genes in Hallmark Inflammatory Response geneset in subcutaneous and visceral adipose tissue of healthy control (Ctrl), cancer weight stable (CWS), and cachectic cancer (CC) patients. The purple boxes delineate inflammatory genes differentially contributing to geneset enrichment in cancer cachexia and controls. The cancer weight stable group demonstrate a similar signal to the cancer cachexia group but the geneset as a whole did not meet statistical significance for enrichment in VAT versus SAT.

We used the Broad Institute Hallmark‐curated genesets collection (17) to examine the data for geneset enrichment. These data are summarised in *Table* 3 and presented in full in the Supporting Information. We first assessed differences between fat depots in each group (*Table* 3). Only Adipogenesis and Fatty Acid metabolism were significantly enriched in all interdepot (VAT compared with SAT) comparisons. In cancer, these genesets were down‐regulated in VAT compared with SAT whilst in the control group the opposite pattern was observed, and they were up‐regulated in VAT compared with SAT (*Table* 3). The Inflammatory response category was increased in expression in both CC and control VAT compared with SAT, but different genes drove the enrichment signal in each group (*Figure* 3). These data suggest a down‐regulation in genes related to growth of adipose tissue in cancer irrespective of weight loss whilst (compared with SAT) inflammation is increased in the VAT of CC patients. We next examined category enrichment in VAT between the groups (Supporting Information). In both CC and CWS VAT, the Adipogenesis and Xenobiotic metabolism (genes encoding proteins involved in the processing of drugs) genesets were decreased in expression compared with control VAT (*Table* 3). Oxidative phosphorylation was significantly down‐regulated in the VAT of CC patients compared with that of controls but was not enriched in the comparison of CWS vs. control VAT (*Table* 3).

**Table 3:** Summary of geneset enrichment analysis

Comparison	Geneset	Direction	FDR
Cachexia VAT vs. SAT	Adipogenesis	Down	2 × 10^−9^
	Fatty Acid Metabolism	Down	0.004
Cancer VAT vs. SAT	Adipogenesis	Down	1 × 10^−6^
	Fatty Acid Metabolism	Down	0.043
Control VAT vs. SAT	Adipogenesis	Up	1.1 × 10^−6^
	Fatty Acid Metabolism	Up	4 × 10^−8^
Cachexia VAT vs. control VAT	Oxidative Phosphorylation	Down	1.24 × 10^−16^
	Adipogenesis	Down	1.24 × 10^−16^
	Fatty Acid Metabolism	Down	3.33 × 10^−9^
	Xenobiotic Metabolism	Down	0.004
Cancer VAT vs. control VAT	Adipogenesis	Down	0.038
	Xenobiotic Metabolism	Down	0.046
Cachexia VAT vs. cancer VAT	Adipogenesis	Down	0.038
	Xenobiotic Metabolism	Down	0.046

FDR, false discovery rate; SAT, subcutaneous adipose tissue; VAT, visceral adipose tissue.

Given our observation of a down‐regulated energy metabolism signature in the VAT of cancer patients, we specifically examined the Gene Ontology category Brown Fat Cell Differentiation (GO:0045444) and found this down‐regulated in VAT in CC (*P* = 5.4 × 10^−6^) and in CWS (*P* = 2 × 10^−7^) compared with control VAT. These data suggest that there is a down‐regulation of mRNA related to adipose expansion and energy use/generation in the VAT of cancer patients and that there is a stronger signature in cachectic cancer patients. We found no evidence of brown fat cell differentiation in this cohort of cachectic cancer patients.

### 3.4 Quantitative polymerase chain reaction gene validation

We used qPCR to examine selected genes including those detected as regulated in the microarray data and the biological processes of fat browning, inflammation, and adipogenesis.

### 3.5 Microarray candidates

There was a significant depot by group interaction effect for ITLN1 (*P* = 0.0006), the gene that encodes for Itln1. ITLN1 was significantly increased in the VAT of the cancer groups (CC *P* = 0.0001, CWS *P* = 0.02) compared with VAT in the control group but was not different between the cancer groups (*P* = 0.5). ITLN1 expression was also significantly greater in VAT compared with SAT in the cancer groups (CC *P* = 0.0004, CWS *P* = 0.03) with no difference in the control group (*P* = 0.9). There were no significant differences in ITLN1 in SAT in any comparison. ALOX15 (a gene that encodes enzymes which act on various polyunsaturated fatty acid substrates) demonstrated a significant depot by group interaction effect (*P* = 0.005) with increased expression in VAT in the cancer groups compared with control (CC *P* = 0.018 and CWS *P* = 0.025, respectively). ALOX15 was increased in VAT compared with SAT in the cancer groups (weight stable *P* = 0.04, cachectic *P* = 0.0046) but not in the control group (*P* = 0.9). These results are in agreement with the microarray results. PPARA2 also demonstrated a depot by group interaction (*P* = 0.004). However multiple testing was unable to detect specific differences. Inspection of the qPCR data suggests lower expression in the VAT of the cancer groups. The direction of change in the qPCR data for the cancer groups reflected the microarray data. PPARA2 expression between the VAT and SAT depot in the control group was significantly different (*P* = 0.03).

### 3.6 Genes involved in fat browning

There were no differences in UCP1 expression in terms of either a depot by group interaction or main effects in agreement with the microarray results. PPARGC1A demonstrated a main effect of depot (*P* = 0.003) with VAT showing higher expression than SAT. Post hoc testing revealed that PPARGC1A was significantly up‐regulated in the VAT compared with SAT in the control group only (*P* = 0.01). The direction of change for PPARGC1A in other comparisons was the same as seen in the microarray data but not statistically significant after multiple testing correction. There were no significant differences in PR/SET domain 16 expression in any comparison.

### 3.7 Inflammation

**Figure 4:**
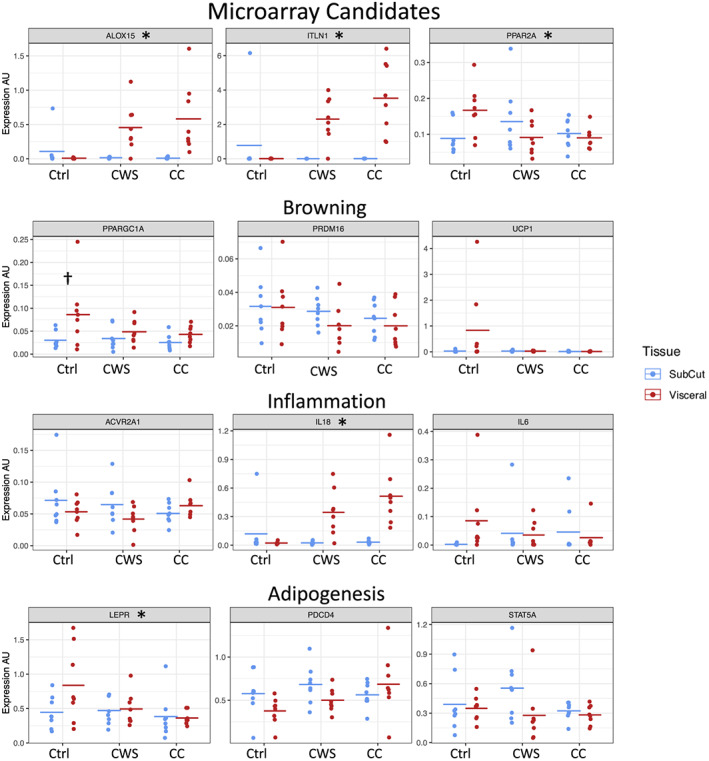
Data from qPCR of selected genes. Categories of genes validated by qPCR were significantly different in microarray data (ALOX15, ITLN1, and PPARA2), involved in browning (PPARGC1A, PRDM16, and UCP1), inflammation related (ACVR2A1, IL6, and IL18), involved in adipogenesis (LEPR, STAT5A, and PDCD4). * = significant group × depot interaction; † = no significant interaction but significant difference between same depot in control group. See main text for full reporting of statistically significant differences. AU, arbitrary units; CC, cancer cachexia; Ctrl, healthy controls; CWS, cancer weight stable.

There were no significant differences in ACVR2A1 expression in terms of interactions or main effects, but the direction of changes seen in the qPCR data agreed with the microarray data. There were no significant differences in IL6 expression in terms of interactions or main effects. IL6 was significantly down‐regulated in CC compared with control VAT in the microarray data. The direction of change was the same in the qPCR data driven by few patients (*Figure* 4) and not statistically significant. IL18 demonstrated a significant depot by group interaction effect (*P* = 0.0009) with increased expression in the VAT of both cancer groups compared with control (CWS *P* = 0.02, CC *P* = 0.0001). Expression of IL18 was also increased in the VAT compared with the SAT in the cancer groups (CWS *P* = 0.03, CC *P* = 0.0006) but not in the control group (*P* = 0.9).

### 3.8 Adipogenesis

LEPR demonstrated a significant depot by group interaction effect (*P* = 0.03) with post hoc testing revealing decreased expression in the VAT in the CC compared with control group (*P* = 0.04). LEPR was also increased in control VAT compared with SAT (*P* = 0.02). STAT5A did not demonstrate any significant interaction or main effects although the effect of fat depot was borderline significant (*P* = 0.06) with specific differences undetectable by post hoc testing. Programmed cell death 4 did not demonstrate any significant interaction or main effects.

### 3.9 ELISA results

**Figure 5:**
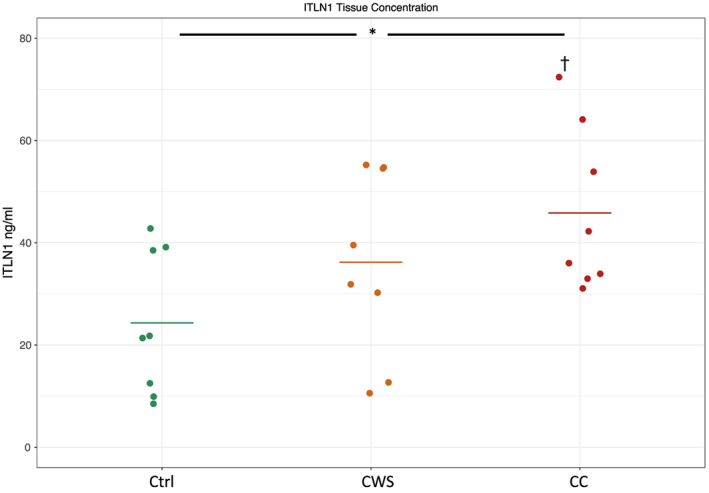
Intelectin‐1 visceral adipose tissue protein concentration (nanogram per millilitre) as measured by enzyme‐linked immunosorbent assay across the control (Ctrl), cancer weight stable (CWS) and cancer cachexia (CC) groups. * = significant effect of group; † = significantly different from control group.

VAT Itln1 concentrations are shown in *Figure* 5. Linear modelling of log Itln1 concentration by group showed a significant effect of the group (*P* = 0.04). There was a significant difference between the CC and control groups (mean Itln1 concentrations 43.6 ng/mL and 20.5 ng/mL respectively, *P* = 0.014) but no statistically significant difference between CWS (mean Itln1 concentration 30.9 ng/mL) and either of the other two groups.

**Figure 6:**
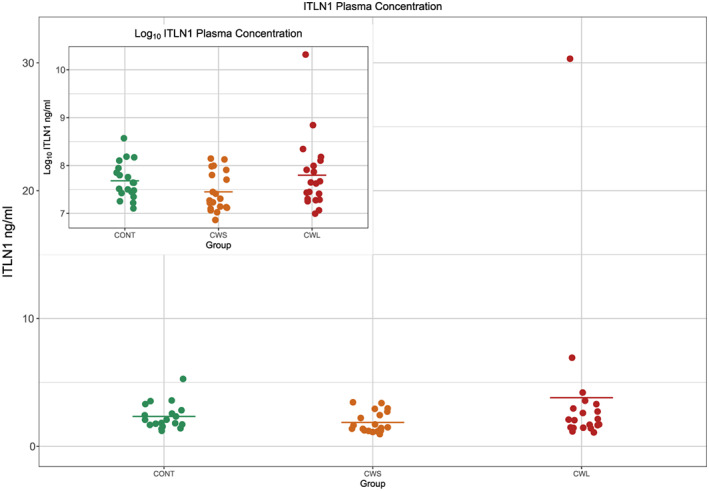
Intelectin‐1 plasma protein concentration (ng/ml) as measured by enzyme‐linked immunosorbent assay across the control (Ctrl), cancer weight stable (CWS) and cancer cachexia (CC) groups. * = significant effect of group; ‡ = significantly different from Ca group.

Given that Itln1 was raised in the VAT of CC patients, we wanted to investigate if it was also raised in the circulating fraction of these patients, and so, plasma ELISA was undertaken. Patients demographics of those included in the plasma ELISA study are summarised in *Table* 4a,b4b. Plasma Itln1 concentrations are shown in *Figure* 6. Mixed effects linear modelling with group as a fixed effect and assay run as a random effect was used to model log Itln1 concentrations. There was a significant effect of group (*P* = 0.028). Post hoc testing using Tukey's correction demonstrated a significant difference in plasma Itln1 concentrations between the CC and CWS groups (mean Itln1 concentrations 2.6 ng/mL and 1.9 ng/mL respectively, *P* = 0.02). There was no significant difference between the control group (mean Itln1 concentration 2.3 ng/mL) and either of the other groups.

**Table 4a:** Demographic details of total patients included in the ITLN‐1 plasma enzyme‐linked immunosorbent assay (ELISA). This cohort includes patients recruited for both array and ELISA studies

Group (n = 20 per group)	Control	Cachexia	Weight stable	P value
Male:Female	14:6	14:6	14:6	/
Age (years)	49 (11)	69 (10)	64 (9)	<0.001
BMI	25.66 (2.81)	25.06 (4.85)	29.93 (7.79)	0.187
%weight loss	0	11.60 (6.58)	0.26 (0.71)	<0.001
SMI	50.69 (7.55)	41.54 (4.82)	55.92 (12.48)	<0.001
VATI	26.23 (20.04)	41.50 (36.65)	111.90 (215.25)	0.008
SATI	52.98 (27.79)	71.85 (59.75)	71.87 (43.75)	0.460

Data are mean (SD).

BMI, body mass index; SATI, subcutaneous adipose index; SMI, skeletal muscle index, VATI, visceral adipose index.

**Table 4b:** Demographic details of additional patients recruited the ITLN‐1 plasma enzyme‐linked immunosorbent assay. Excludes those patients recruited to the microarray study

Group (*n* = 12)	Control	Cachexia	Weight stable	*P* value
Male:Female	10:2	9:3	8:4	/
Age (years)	48 (14)	69 (10)	68 (10)	<0.001
BMI	25.77 (2.67)	25.35 (3.68)	29.05 (7.61)	0.697
%weight loss	0	11.21 (6.78)	0.43 (0.90)	<0.001
SMI	51.42 (7.97)	42.56 (4.84)	56.04 (14.74)	0.019
VATI	23.65 (15.11)	51.41 (40.68)	151.78 (279.69)	0.016
SATI	51.24 (19.89)	74.69 (60.69)	69.77 (45.06)	0.620

BMI, body mass index; SATI, subcutaneous adipose index; SMI, skeletal muscle index, VATI, visceral adipose index.

## 4 Discussion

Different mechanisms have been previously proposed to account for the changes in adipose tissue seen in cachexia. In CC, human and animal studies have shown increased rates of lipid mobilisation and loss of adipose tissue before any demonstrable changes in loss of muscle mass.^30^ An increased rate of adipose tissue loss has also been associated with worsening prognosis in cancer patients highlighting the importance of gaining a greater understanding of the mechanisms involved. To the best of our knowledge, this is the first study to examine both SAT and VAT depots in CC patients. We have shown that SAT and VAT have unique gene expression signatures, and VAT has an altered gene expression signature in cancer. We found increased expression of Itln1 and Intelectin‐2 in particular suggesting these adipokines may play a role in adipose changes in response to cancer. Itln1 may be a target for therapeutic manipulation.

The gene which showed the largest difference in expression was ITLN1; a 34 kDa novel adipokine. Expression of ITLN2 was also high but not as great as ITLN1 and so ITLN1 formed the focus of our study. ITLN1 is preferentially produced by stromovascular cells in VAT^31^ but has also been identified in human epicardial fat cells, mesothelial cells, airway goblet cells, and cells lining the gut and ovaries.^32^ A series of studies have linked raised levels of ITLN1 to various cancers.^33^, ^34^, ^35^, ^36^, ^37^, ^38^, ^39^, ^40^ ITLN1 has been suggested to promote cancer cell growth by triggering genomic instability via phosphatidylinositol‐3 kinase downstream effector signalling pathways.^41^ The role of ITLN1 in CC however, has not been well characterised. Randomized control trials have shown that weight loss significantly increases plasma ITLN1 concentration^42^ whereas hyper‐insulinaemic induction in healthy individuals reduces ITLN1 plasma concentration.^43^ Decreased levels of ITLN1 have been associated with obesity in patients with ovarian cancer,^43^ and levels of ITLN1 measured before patients were diagnosed with colorectal cancer have been confined to nonobese individuals suggesting it has a role in weight loss.^44^

Our microarray results confirmed that expression of ITLN1 was higher in VAT in cancer although not confined to CC patients. Plasma levels of Itln1 were significantly increased in CC compared with CWS groups but showed no difference with controls suggesting it possibly has a role in cancer induced weight loss. However, as in the previous studies, we found that plasma Itln1 differences were driven by a few individuals with high levels. High interindividual variability in plasma ITLN1 likely limited the ability to detect a statistically significant effect across all patient groups. Larger patient studies will be required in the future to ascertain any true difference in circulating ITLN1 in cancer. The significant increase in intelectin seen in the VAT ELISA of cachectic patients suggests a possible role in the pathogenesis of cachexia. There was a clear increasing gradient from controls through CWS to CC in VAT Itln1 level. This was not reflected in the plasma Itln1 protein levels. These results are similar to those we have previously seen with zinc a‐glycoprotein [ZAG]^6^ in CC. This pattern of increased tissue protein levels but no change in circulating levels could be explained by cells outside adipose tissue contributing to circulating protein. Alternatively, there could be a predominantly paracrine role for adipose tissue secreted protein that is not released into the circulation.

We found no evidence of increased browning in white adipose tissue in our cohort. Previously increased thermogenic activity of brown and beige adipose tissue has been shown to contribute to the increased energy expenditure and weight loss in rodent models of cachexia.^45^, ^46^, ^47^ White adipose tissue browning is associated with increased expression of UCP1, which uncouples mitochondrial respiration towards thermogenesis instead of adenosine triphosphate synthesis, leading to increased lipid mobilization and energy expenditure.^48^ Whilst browning of white fat depots contributes to futile energy cycling in cachexia in animal models the importance of this is uncertain.^49^, ^50^ Genes involved in fat browning in the present study were not up‐regulated suggesting browning has a limited role in the pathogenesis of cachexia in the recruited patients and highlights the importance of patient‐based research. These data provide the basis for future studies that further examine the role of adipose biology in the metabolic effects of cancer in humans.

Cachexia is thought to be a chronic inflammatory state, and changes in inflammatory mediators in adipocytes may be capable of inducing changes in adipose tissue homeostasis.^51^ Several studies have shown adipose tissue to be the target of several pro‐cachectic factors as well as adipose tissue itself being an important source of inflammatory mediators.^52^, ^53^, ^54^, ^55^, ^56^ The majority of studies assessing inflammation in cachexia have examined inflammatory markers from a systemic point of view rather than a tissue‐specific one with IL6 in particular frequently found to be raised in the plasma of CC patients.^57^ IL6 was down‐regulated in adipose tissue in CC suggesting a possible homeostatic response. Specific adipose tissue inflammation is important to consider as it may be an event very early on in the cachectic process prior to any systemic effects being demonstrated.

Adipogenesis is the process by which preadipocytes differentiate into mature adipocytes able to store triglycerides and secrete adipokines. Some studies have shown that adipogenic genes are down‐regulated in animal epididymal and retroperitoneal fat in cachexia.^54^, ^58^ Co‐culture studies have demonstrated inhibition of pre‐adipocyte maturation in the presence of tumour cells that was associated with increased inflammatory cytokines.^59^ Changes in adipogenesis are likely to precede the clinical signs of fat wasting and tissue inflammation. It is possible therefore that factors such as increased infiltration of macrophages and production of inflammatory cytokines silence adipogenic genes and/or increase lipolysis leading to adipose wasting in cachexia.

The current study confers similar limitations to other microarray studies most notably in the small sample size. However exploratory work of this nature is valuable as it provides testable hypotheses to be taken forward. Another limitation of this study was the availability of omental and perinephric adipose tissue in the cancer and control groups, respectively. Differences in these two depots are not well defined. In rodents, there are possible depot‐specific differences in innervation, but this has not been documented in humans.^60^ A very small study in humans has suggested blood flow in omental fat may be higher than in perinephric fat though the difference was not significant.^61^ This may potentially the affect removal of lipolytic products and the rates of lipolysis. The patients studied were predominantly male, and we are therefore unable to draw conclusions about sex‐specific differences. There was no longitudinal follow‐up of patients done, and so, it is unclear whether the patients in the CWS group went on to develop cachexia and therefore may have been pre‐cachectic at the time of biopsy. We also excluded patients in the control and CWS groups who were sarcopenic on CT scan. In doing so, we have assumed that a reduction in SMI in the cachectic group was because of disease. Given that these patients have a median age of 70, it is possible that some were sarcopenic prior to the start of the disease process. We chose to define patients based on this specific definition of cancer cachexia (>2% weight loss and low muscularity) as it is the previous definition that has been associated with histological wasting.^16^ Notably, all but one of the participants in our study defined as having cancer cachexia had >5% weight loss (over 6 months). Reanalysis of the ITLN1 qPCR data after removal of this single subject did not affect the conclusions of that analysis.

## 5 Conclusions

This exploratory analysis confirms differential gene expression in adipose depots in patients with cancer with and without cachexia and highlights the importance of VAT in cancer. VAT may have a fundamental role in cachexia, but the down‐regulation of energy metabolism genes implies a limited role for browning in cachectic patients in our cohort as suggested in pre‐clinical models. Future mechanistic studies are important to evaluate the effects of VAT in cachexia and in particular the role of Itln1.

## Funding

J. M. is supported by Cancer Research UK and the Royal College of Surgeons of Edinburgh. R. J. E. S. is supported by a NHS Research for Scotland (NRS)‐funded post.

## Ethical standards

The study conforms to the declaration of Helsinki. All patients provided written, informed consent. The manuscript complies with the ethical guidelines for authorship and publishing in the *Journal of Cachexia, Sarcopenia and Muscle*.^62^

## Conflict of interest

The authors declare no conflicts of interest.

## Supporting information

Data S1. Supporting Information

Data S2. Supporting Information

## References

[1] Fearon KCH . Cancer cachexia and fat‐muscle physiology. N Engl J Med 2011;365:565–567. 21830971 10.1056/NEJMcibr1106880

[2] Fearon K , Strasser F , Anker SD , Bosaeus I , Bruera E , Fainsinger RL , et al. Definition and classification of cancer cachexia: an international consensus. Lancet Oncol 2011;12:489–495. 21296615 10.1016/S1470-2045(10)70218-7

[3] Ebadi M , Mazurak VC . Evidence and mechanisms of fat depletion in cancer. Nutrients 2014;6:5280–5297. 25415607 10.3390/nu6115280 PMC4245589

[4] Tsoli M , Swarbrick MM , Robertson GR . Lipolytic and thermogenic depletion of adipose tissue in cancer cachexia. Semin Cell Dev Biol, Mechanisms of cancer cachexiaMeiosis and Recombination 2016;54:68–81. 26529279 10.1016/j.semcdb.2015.10.039

[5] Rydén M , Agustsson T , Laurencikiene J , Britton T , Sjölin E , Isaksson B , et al. Lipolysis—not inflammation, cell death, or lipogenesis—is involved in adipose tissue loss in cancer cachexia. Cancer 2008;113:1695–1704. 18704987 10.1002/cncr.23802

[6] Mracek T , Stephens NA , Gao D , Bao Y , Ross JA , Ryden M , et al. Enhanced ZAG production by subcutaneous adipose tissue is linked to weight loss in gastrointestinal cancer patients. Br J Cancer 2011;104:441–447. 21245862 10.1038/sj.bjc.6606083 PMC3049573

[7] Silverio R , Lira F , Oyama LM , Oller Do Nascimento CM , Otoch JP , Alcantara PSM , et al. Lipases and lipid droplet‐associated protein expression in subcutaneous white adipose tissue in cachectic patients with cancer. Lipids Health Dis 2017;16:159. 28830524 10.1186/s12944-017-0547-x PMC5568087

[8] Dahlman I , Mejhert N , Linder K , Augustsson T , Mutch DM , Kulyte A , et al. Adipose tissue pathways involved in weight loss of cancer cachexia. Br J Cancer 2010;102:1541–1548. 20407445 10.1038/sj.bjc.6605665 PMC2869165

[9] Ebadi M , Baracos VE , Bathe OF , Robinson LE , Mazurak VC . Loss of visceral adipose tissue precedes subcutaneous adipose tissue and associates with N‐6 fatty acid content. Clin Nutr 2016;35:1347–1353. 26972089 10.1016/j.clnu.2016.02.014

[10] Modesitt SC , Hsu JY , Chowbina SR , Lawrence RT , Hoehn KL . Not all fat is equal: differential gene expression and potential therapeutic targets in subcutaneous adipose, visceral adipose, and endometrium of obese women with and without endometrial cancer. Int J Gynecol Canc: Official Journal of the International Gynecological Cancer Society 2012;22:732–741. 10.1097/IGC.0b013e3182510496 22635025

[11] Das SK , Eder S , Schauer S , Diwoky C , Temmel H , Guertl B , et al. Adipose triglyceride lipase contributes to cancer‐associated cachexia. Science [New York, NY] 2011;333:233–238. 10.1126/science.1198973 21680814

[12] Gallagher IJ , Jacobi C , Tardif N , Rooyackers O , Fearon K . Omics/systems biology and cancer cachexia. Semin Cell Dev Biol 2016;54:92–103. 26783720 10.1016/j.semcdb.2015.12.022

[13] Stephens N , Gallagher IJ , Rooyackers O , Skipworth RJ , Tan BH , Marstrand T , et al. Using transcriptomics to identify and validate novel biomarkers of human skeletal muscle cancer cachexia. Genome Med 2010;2:1. 20193046 10.1186/gm122 PMC2829926

[14] Gallagher IJ , Stephens NA , MacDonald AJ , Skipworth RJE , Husi H , Greig C , et al. Suppression of skeletal muscle turnover in cancer cachexia: evidence from the transcriptome in sequential human muscle biopsies. Clin Cancer Res 2012;18:2817–2827. 22452944 10.1158/1078-0432.CCR-11-2133

[15] Martin L , Bridsell L , MacDonald N , Reiman T , Clandinin MT , McCargar LJ , et al. Cancer cachexia in the age of obesity: skeletal muscle depletion is a power prognostic factor independent of body mass index. J Clin Oncol 2013;31:1539–1547. 23530101 10.1200/JCO.2012.45.2722

[16] Johns N , Hatakeyama S , Stephens NA , Degen M , Degen S , Frieauff W , et al. Clinical classification of cancer cachexia: phenotypic correlates in human skeletal muscle. PLoS ONE 2014;9:e83618. 24404136 10.1371/journal.pone.0083618 PMC3880262

[17] Wang X , Spandidos A , Wang H , Seed B . PrimerBank: a PCR primer database for quantitative gene expression analysis, 2012 update. Nucleic Acids Res 2012 Jan;40:D1144–D1149. 22086960 10.1093/nar/gkr1013 PMC3245149

[18] Ye J , Coulouris G , Zaretskaya I , Cutcutache I , Rozen S , Madden TL . Primer‐BLAST: a tool to design target‐specific primers for polymerase chain reaction. BMC Bioinformatics 2012 Jun 18;13:134. 22708584 10.1186/1471-2105-13-134 PMC3412702

[19] Bustin SA , Benes V , Garson JA , Hellemans J , Huggett J , Kubista M , et al. The MIQE guidelines: minimum information for publication of quantitative real‐time PCR Experiments. Clin Chem 2009;55:611–622. 19246619 10.1373/clinchem.2008.112797

[20] Perkins JR , Dawes JM , McMahon SB , Bennett DHL , Orengo C , Kohl M . ReadqPCR and NormqPCR: R packages for the reading, quality checking and normalization of RT‐qPCR quantification cycle [Cq] data. BMC Genomics 2012;13:296. 22748112 10.1186/1471-2164-13-296 PMC3443438

[21] Vandesompele J , De Preter K , Pattyn F , Poppe B , Van Roy N , De Paepe A , et al. Accurate normalization of real‐time quantitative RT‐PCR data by geometric averaging of multiple internal control genes. Genome Biol 2002;3:1. 10.1186/gb-2002-3-7-research0034 PMC126239 12184808

[22] Dai M , Wang P , Boyd AD , Kostov G , Athey B , Jones EG , et al. Evolving gene/transcript definitions significantly alter the interpretation of GeneChip data. Nucleic Acids Res 2005;33:e175. 16284200 10.1093/nar/gni179 PMC1283542

[23] Piccolo SR , Sun Y , Campbell JD , Lenburg ME , Bild AH , Johnson WE . A single‐sample microarray normalization method to facilitate personalized‐medicine workflows. Genomics 2012;100:337–344. 22959562 10.1016/j.ygeno.2012.08.003 PMC3508193

[24] Gentleman R , Carey VJ , Bates DM , Bolstad B , Dettling M , Dudoit S , et al. Bioconductor: open software development for computational biology and bioinformatics. Genome Biol 2004;5:R80. 15461798 10.1186/gb-2004-5-10-r80 PMC545600

[25] Wu D , Smyth GK . Camera: a competitive gene set test accounting for inter‐gene correlation. Nucleic Acids Res 2012;40:e133. 22638577 10.1093/nar/gks461 PMC3458527

[26] Liberzon A , Birger C , Thorvaldsdottir MG , Mesirov JP , Tamayo P . The molecular signatures database hallmark geneset collection. Cell Systems 2015;1:417–425. 26771021 10.1016/j.cels.2015.12.004 PMC4707969

[27] Bates D , Machler M , Bolker B , Walker S . Fitting linear mixed effects models using lme4. J Stat Softw 2015;67:1–47.

[28] https://cran.r‐project.org/web/packages/emmeans/emmeans.pdf accessed March 2019

[29] Fong Y , Sebestyen K , Yu X , Gilbert P , Self S . nCal: an R package for non‐linear calibration. Bioinformatics 2013;29:2653–2654. 23926226 10.1093/bioinformatics/btt456 PMC3789552

[30] Agustsson T , Wikrantz P , Rydén M , Brismar T , Isaksson B . Adipose tissue volume is decreased in recently diagnosed cancer patients with cachexia. Nutrition 2012;28:851–855. 22480800 10.1016/j.nut.2011.11.026

[31] Yang RZ , Lee MJ , Hu H , Pray J , Wu HB , Hansen BC , et al. Identification of omentin as a novel depot‐specific adipokine in human adipose tissue: possible role in modulating insulin action. Am J Physiol Endocrinol Metab 2006;290:E1253–E1261. 16531507 10.1152/ajpendo.00572.2004

[32] Watanabe T , Watanabe‐Kominato K , Takahashi Y , Kojima M , Watanabe R . Adipose tissue‐derived Omentin‐1 function and regulation. Compr Physiol 2017;7:765–781. 28640441 10.1002/cphy.c160043

[33] Tsuji S , Tsuura Y , Morohoshi T , Shinohara T , Oshita F , Yamada K , et al. Secretion of intelectin‐1 from malignant pleural mesothelioma into pleural effusion. Br J Cancer 2010;103:517–523. 20628387 10.1038/sj.bjc.6605786 PMC2939784

[34] Kim HJ , Kang UB , Lee H , Jung JH , Lee ST , Yu MH , et al. Profiling of differentially expressed proteins in stage IV colorectal cancers with good and poor outcomes. J Proteomics 2012;75:2983–2997. 22178445 10.1016/j.jprot.2011.12.002

[35] Zheng L , Weng M , Qi M , Qi T , Tong L , Hou X , et al. Aberrant expression of intelectin‐1 in gastric cancer: its relationship with clinicopathological features and prognosis. J Cancer Res Clin Oncol 2012;138:163–172. 22083213 10.1007/s00432-011-1088-8 PMC11824276

[36] Washimi K , Yokose T , Yamashita M , Kageyama T , Suzuki K , Yoshihara M , et al. Specific expression of human intelectin‐1 in malignant pleural mesothelioma and gastrointestinal goblet cells. PLoS ONE 2012;7:e39889. 22768319 10.1371/journal.pone.0039889 PMC3388067

[37] Uyeturk U , Sarıcı H , Kın TB , Eroglu M , Kemahlı E , Uyeturk U , et al. Serum omentin level in patients with prostate cancer. Med Oncol 2014;31:923. 24659266 10.1007/s12032-014-0923-6

[38] Li D , Mei H , Pu J , Xiang X , Zhao X , Qu H , et al. Intelectin 1 suppresses the growth, invasion and metastasis of neuroblastoma cells through up‐regulation of N‐myc downstream regulated gene 2. Mol Cancer 2015;14:47. 25889839 10.1186/s12943-015-0320-6 PMC4359454

[39] Li D , Zhao X , Xiao Y , Mei H , Pu J , Xiang X , et al. Intelectin 1 suppresses tumor progression and is associated with improved survival in gastric cancer. Oncotarget 2015;6:16168–16182. 25965823 10.18632/oncotarget.3753 PMC4599263

[40] Shen XD , Zhang L , Che H , Zhang YY , Yang C , Zhou J , et al. Circulating levels of adipocytokine omentin‐1 in patients with renal cell cancer. Cytokine 2016;77:50–55. 26539805 10.1016/j.cyto.2015.09.004

[41] Wu SS , Liang QH , Liu Y , Cui RR , Yuan LQ , Liao EY . Omentin‐1 stimulates human osteoblast proliferation through PI3K/Akt signal pathway. Int J Endocrinol 2013;2013:368970. 23606838 10.1155/2013/368970 PMC3626246

[42] Moreno‐Navarrete JM , Catalan V , Ortega F , Gomez‐Ambrosi J , Ricart W , Fruhbeck G , et al. Circulating omentin concentration increases after weight loss. Nutr Metab 2010;7:27. 10.1186/1743-7075-7-27 PMC2859768 20380714

[43] Tan BK , Adya R , Farhatullah S , Lewandowski KC , O'Hare P , Lehnert H , et al. Omentin‐1, a novel adipokine, is decreased in overweight insulin‐resistant women with polycystic ovary syndrome: ex vivo and in vivo regulation of omentin‐1 by insulin and glucose. Diabetes 2008;57:801–808. 18174521 10.2337/db07-0990

[44] Aleksandrova K , Nimptsch K , Pischon T . Influence of obesity and related metabolic alterations on colorectal cancer risk. Curr Nutr Rep 2013;2:1–9. 23396857 10.1007/s13668-012-0036-9 PMC3562548

[45] Kir S , White JP , Kleiner S , Kazak L , Cohen P , Baracos VE , et al. Tumour‐derived PTH‐related protein triggers adipose tissue browning and cancer cachexia. Nature 2014;513:100–104. 25043053 10.1038/nature13528 PMC4224962

[46] Petruzzelli M , Schweiger M , Schreiber R , Campos‐Olivas R , Tsoli M , Allen J , et al. A switch from white to brown fat increases energy expenditure in cancer‐associated cachexia. Cell Metab 2014;20:433–447. 25043816 10.1016/j.cmet.2014.06.011

[47] Tsoli M , Moore M , Burg D , Painter A , Taylor R , Lockie SH , et al. Activation of thermogenesis in brown adipose tissue and dysregulated lipid metabolism associated with cancer cachexia in mice. Cancer Res 2012;72:4372–4382. 22719069 10.1158/0008-5472.CAN-11-3536

[48] Crichton PG , Lee Y , Kunji ERS . The molecular features of uncoupling protein 1 support a conventional mitochondrial carrier‐like mechanism. Biochimie 2017;134:35–50. 28057583 10.1016/j.biochi.2016.12.016 PMC5395090

[49] Park A , Kim WK , Bae KH . Distinction of white, beige and brown adipocytes derived from mesenchymal stem cells. World J Stem Cells 2014;6:33–42. 24567786 10.4252/wjsc.v6.i1.33 PMC3927012

[50] Sacks H , Symonds ME . Anatomical locations of human brown fat tissue. Diabetes 2013;62:1783–1790. 23704519 10.2337/db12-1430 PMC3661606

[51] DiSpirito JR , Mathis D . Immunological contributions to adipose tissue homestasis. Semin Immunol 2015;27:315–321. 26616665 10.1016/j.smim.2015.10.005 PMC4681639

[52] Batista ML Jr , Neves RX , Peres SB , Yamashita AS , Shida CS , Farmer SR , et al. Heterogeneous time‐dependent response of adipose tissue during the development of cancer cachexia. J Endocrinol 2012;215:363–373. 23033362 10.1530/JOE-12-0307

[53] Argiles JM , Stemmler B , Lopez‐Soriano FJ , Busquets S . Inter‐tissue communication in cancer cachexia. Nat Rev Endocrinol 2018;15:9–20. 30464312 10.1038/s41574-018-0123-0

[54] Batista ML , Olivan M , Alcantara PS , Sandoval R , Peres SB , Neves RX , et al. Adipose tissue‐derived factors as potential biomarkers in cachectic cancer patients. Cytokine 2013;61:532–539. 23200412 10.1016/j.cyto.2012.10.023

[55] Alves MJ , Figuerêdo RG , Azevedo FF , Cavallaro DA , Neto NI , Lima JD , et al. Adipose tissue fibrosis in human cancer cachexia: the role of TGFB pathway. BMC Cancer 2017;17:190. 28288584 10.1186/s12885-017-3178-8 PMC5348844

[56] Batista ML , Henriques FS , Neves RX , Olivian MR , Matos‐Neto EM , Alcantara PSM , et al. Cachexia‐associated adipose tissue morphological rearrangement in gastrointestinal cancer patients. J Cachexia Sarcopenia Muscle 2016;7:37–47. 27066317 10.1002/jcsm.12037 PMC4799865

[57] White JP . IL‐6, cancer and cachexia: metabolic dysfunction creates the perfect storm. Transl Cancer Res 2017;6:280–285. 10.21037/tcr.2017.03.52 PMC6372111 30766805

[58] Bing C , Russell S , Becket E , Pope M , Tisdale MJ , Trayhurn P , et al. Adipose atrophy in cancer cachexia: morphologic and molecular analysis of adipose tissue in tumour‐bearing mice. BJC 2006;95:1028–1037. 17047651 10.1038/sj.bjc.6603360 PMC2360696

[59] Lopes MA , Franco FO , Henriques F , Peres SB , Batista ML Jr . Effects of LLC tumoral secretory products in coculture system on adipocyte differentiation. J Cachexia Sarcopenia Muscle 2015;6:398–509.

[60] Lee MJ , Wu Y , Fried SK . Adipose tissue heterogeneity: implication of depot differences in adipose tissue for obesity complications. Mol Aspects Med 2013;34:1–11. 23068073 10.1016/j.mam.2012.10.001 PMC3549425

[61] Virtanen KA , Lonnroth P , Parkkola R , Peltoniemi P , Asola M , Viljanen T , et al. Glucose uptake and perfusion in subcutaneous and visceral adipose tissue during insulin stimulation in nonobese and obese humans. J Clin Endocrinol Metab 2002 Aug;87:3902–3910. 12161530 10.1210/jcem.87.8.8761

[62] von Haehling S , Morley JE , Coats AJS , Anker SD . Ethical guidelines for publishing in the Journal of Cachexia, Sarcopenia and Muscle: update 2019. J Cachexia Sarcopenia Muscle 2019; 10: 1143‐1145. 31661195 10.1002/jcsm.12501 PMC6818444

