# Supplementary material for: Adipose depot gene expression and intelectin‐1 in the metabolic response to cancer and cachexia
Source: J Cachexia Sarcopenia Muscle. 2020 Mar 31;11(4):1141–53. doi: 10.1002/jcsm.12568 (PMC7432578; doi:10.1002/jcsm.12568)
Supplement: Supplementary file 2 — Data S2. Supporting Information [file JCSM-11-1141-s002.docx]

Full microarray differential expression analysis and category enrichment results.
